# Rad9, Rad17, TopBP1 and Claspin Play Essential Roles in Heat-Induced Activation of ATR Kinase and Heat Tolerance

**DOI:** 10.1371/journal.pone.0055361

**Published:** 2013-02-01

**Authors:** Munkhbold Tuul, Hiroyuki Kitao, Makoto Iimori, Kazuaki Matsuoka, Shinichi Kiyonari, Hiroshi Saeki, Eiji Oki, Masaru Morita, Yoshihiko Maehara

**Affiliations:** 1 Department of Molecular Oncology, Graduate School of Medical Sciences, Kyushu University, Fukuoka, Japan; 2 Department of Surgery and Science, Graduate School of Medical Sciences, Kyushu University, Fukuoka, Japan; 3 Innovative anticancer strategy for therapeutics and diagnosis group, Innovation Center for Medical Redox Navigation, Kyushu University, Fukuoka, Japan; 4 Tokushima Research Center, Taiho Pharmaceutical Co., Ltd., Tokushima, Japan; North Carolina State University, United States of America

## Abstract

Hyperthermia is widely used to treat patients with cancer, especially in combination with other treatments such as radiation therapy. Heat treatment *per se* activates DNA damage responses mediated by the ATR-Chk1 and ATM-Chk2 pathways but it is not fully understood how these DNA damage responses are activated and affect heat tolerance. By performing a genetic analysis of human HeLa cells and chicken B lymphoma DT40 cells, we found that heat-induced Chk1 Ser345 phosphorylation by ATR was largely dependent on Rad9, Rad17, TopBP1 and Claspin. Activation of the ATR-Chk1 pathway by heat, however, was not associated with FancD2 monoubiquitination or RPA32 phosphorylation, which are known as downstream events of ATR kinase activation when replication forks are stalled. Downregulation of *ATR, Rad9*, *Rad17, TopBP1* or *Claspin* drastically reduced clonogenic cell viability upon hyperthermia, while gene knockout or inhibition of ATM kinase reduced clonogenic viability only modestly. Suppression of the ATR-Chk1 pathway activation enhanced heat-induced phosphorylation of Chk2 Thr68 and simultaneous inhibition of ATR and ATM kinases rendered severe heat cytotoxicity. These data indicate that essential factors for activation of the ATR-Chk1 pathway at stalled replication forks are also required for heat-induced activation of ATR kinase, which predominantly contributes to heat tolerance in a non-overlapping manner with ATM kinase.

## Introduction

Hyperthermia is one of the oldest methods used to treat cancer patients. When hyperthermia is combined with other treatments, a significant improvement in the clinical outcome is observed [1]. We have used hyperthermia together with chemoradiotherapy to treat patients with esophageal cancer and rectal cancer with clinical benefit [2], [3]. Currently, heat is one of the most potent sensitizers to the action of ionizing radiation (IR) in cells and in human tumors [4], but how heat enhances tumor cytotoxicity is not fully understood.

One possibility is that heat induces DNA damage. DNA degradation was detected in heat-treated Chinese hamster ovary cells by the alkaline elution method [5]. DNA strand scissions were detected as early as 15 minutes in heat-treated HeLa cells in an *in situ* nick translation assay, and the heat-induced DNA scissions were closely correlated with cytotoxicity [6]. These results suggest that DNA single-strand breaks or gaps are induced by heat. Heat also induces the phosphorylation and nuclear foci formation of histone H2AX at Ser139 (γH2AX) [7], [8], [9]. In many cases, γH2AX nuclear foci are indicators of DNA double-strand breaks (DSBs) [10] and γH2AX plays a critical role in the recruitment of repair factors to sites of DNA damage [11]. Heat-induced γH2AX nuclear foci have been suggested to coincide with heat-induced DNA DSBs, which cause the loss of cell viability [7], [8]. Another report showed that DNA DSBs are not associated with heat-induced γH2AX nuclear foci, because the recruitment of DSB repair factors such as 53BP1 and SMC1 was not observed [9].

Heat *per se* induces several steps associated with DNA damage responses (DDR). Heat induces the autophosphorylation of ATM at Ser1981 and activates its kinase activity, but this occurs in the absence of apparent DNA strand breaks [9]. Prior ATM activation by heat may interfere with the normal DDR induced by IR, which is required for the activation of cell cycle checkpoints and chromosomal DNA DSB repair. Indeed, heat perturbs IR-induced DDR mediated by 53BP1 and its downstream targets, which may explain heat radiosensitization [12]. Heat-induced alterations in chromatin structure cause aberrant activation of DDR and reduce accessibility of DNA repair machinery to the damage sites of the following IR [4]. Recently, the ATR-Chk1 pathway was shown to be preferentially activated by heat [13]. Selective inhibitors of ATR or Chk1 enhanced heat-induced apoptosis, and their effect was more prominent than selective inhibitors of ATM or Chk2, suggesting the importance of the ATR-Chk1 pathway in protecting cells from heat cytotoxicity. The ATR-Chk1 pathway is activated when replication forks are stalled [14], and various factors, including replication protein A (RPA)-coated single-strand DNA (ssDNA), 5′ ends at primer-template junctions, ATR interacting protein (ATRIP), TopBP1, Claspin, polymerase alpha, Rad9-Rad1-Hus1 (9-1-1) heterotrimeric clamp and Rad17-RFC clamp loader of 9-1-1, are involved in this process [15]. ATR kinase phosphorylates multiple downstream targets other than Chk1, such as RPA32 [16] and FancI [17], [18], which play an important role in S phase checkpoint and Fanconi anemia (FA) pathway activation, respectively. However, it is not known which factors are required for heat-induced activation of the ATR-Chk1 pathway or which downstream targets of ATR kinase are phosphorylated at high temperature.

To understand the mechanism for heat-induced activation of the signaling pathways belonging to ATR-Chk1 and ATM-Chk2 axes, we performed genetic analysis using human HeLa cells and chicken DT40 cells. We found that heat-induced activation of the ATR-Chk1 pathway was largely dependent on Rad9, Rad17, TopBP1 or Claspin, essential factors for activation of ATR-Chk1 pathway at stalled replication forks. Heat-induced activation of the ATR-Chk1 pathway, however, was not associated with FancD2 monoubiquitination, an indicator of FA pathway activation [19], or RPA32 phosphorylation [16], which suggests that heat does not activate all downstream targets of ATR kinase. ATR and ATM kinases contributed to heat tolerance in a non-overlapping manner and simultaneous inhibition of ATR and ATM kinases with caffeine significantly enhanced the cytotoxic effect of hyperthermia. This study revealed the evolutionarily conserved roles of heat-induced activation of DNA damage response.

## Results

### Heat induction of Chk1 phosphorylation but not of FancD2 monoubiquitination in HeLa cells and chicken DT40 cells

To analyze cellular responses to heat, HeLa and chicken B lymphoma DT40 cells and their mutants were used as model systems. A temperature of 5.5°C above the normal culture temperature (42.5°C for HeLa cells, 45°C for DT40 cells, normal culture temperature for HeLa cells and DT40 cells is 37°C and 39.5°C, respectively) was used to provoke hyperthermia, because this temperature induces cell death via disruption of DNA repair machinery [8].

**Figure 1:**
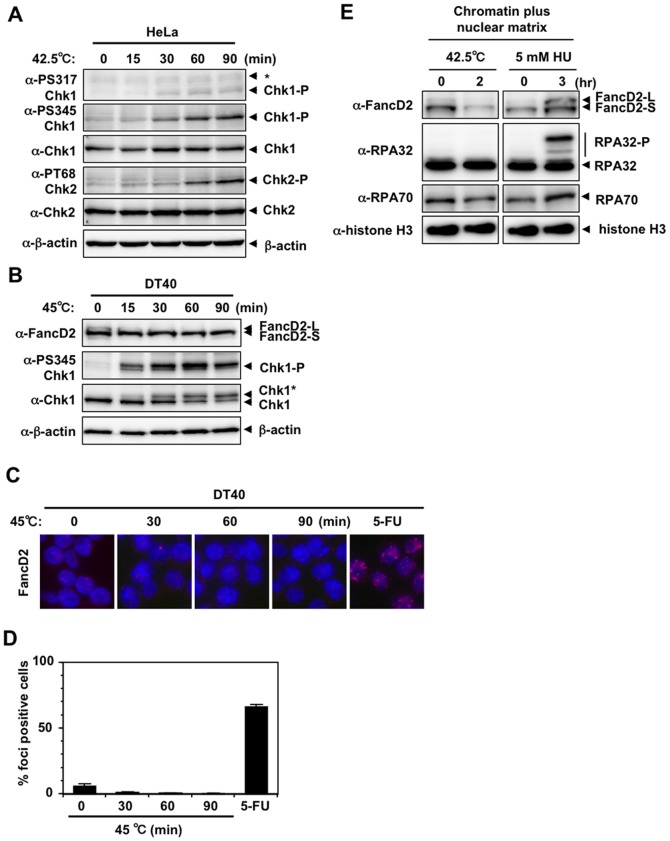
DNA damage response by heat stress. **A.** Western blot. HeLa cells were cultured at 42.5°C for the indicated time. Non-specific bands were indicated as *. **B.** Western blot. Wild-type DT40 cells were cultured at 45°C for the indicated time. **C.** Nuclear foci of FancD2. Wild-type DT40 cells were cultured at 45°C for the indicated time. Wild-type DT40 cells cultured in the presence of 200 µM 5-FU for 16 hours are shown as a positive control (5-FU) [23]. **D.** The percentage of FancD2 nuclear foci-positive cells in **C** is shown. **E.** Subcellular fractionation of HeLa cells cultured at 42.5°C for 2 hours or at 37°C in the presence of 5 mM hydroxyurea (HU) for 3 hours. Chromatin plus nuclear matrix fraction was isolated as described in Materials and Methods. Ten µg (FancD2, RPA70 and RPA32) or 2 µg (histone H3) of protein were subjected to SDS-PAGE and Western blot.

As reported previously [13], phosphorylation of Chk1 Ser317 and Ser345 and Chk2 Thr68, the primary targets of ATR and ATM kinases, respectively, was induced when HeLa cells were incubated at 42.5°C (Fig. 1A). Chk1 Ser317 and Ser345 phosphorylation was detected as early as 30 minutes after the shift to 42.5°C, whereas phosphorylation of Chk2 Thr68 was detected at 60 minutes (Fig. 1A). In DT40 cells, Chk1 Ser345 phosphorylation was detected as early as 15 minutes after the shift to 45°C (Fig. 1B). In addition, slower migrating forms of Chk1 (indicated as Chk1* in Fig. 1B), indicating its posttranslational modification, were induced with similar kinetics (Fig. 1B). However, monoubiquitination of FancD2 (Fig. 1B) or FancD2 nuclear foci (Fig. 1C and 1D) were not induced by heat in DT40 cells. Furthermore, induction of FancD2 monoubiquitination, RPA32 phosphorylation or RPA70/RPA32 protein accumulation was not detected in the chromatin plus nuclear matrix fraction of heat-treated HeLa cells, while such induction was clearly detected in the chromatin plus nuclear matrix fraction of hydroxyurea (HU)-treated HeLa cells (Fig. 1E). This result suggests that not all downstream events of ATR kinase were induced by heat.

### Rad9- and Rad17-deficiency suppressed heat-induced Chk1 Ser345 phosphorylation and enhanced heat cytotoxicity

The 9-1-1 clamp and the Rad17-RFC clamp loader play essential roles in activation of the ATR-Chk1 pathway at stalled replication forks [14], [20]. We examined the possible involvement of Rad9 and Rad17 in the heat-induced ATR-Chk1 pathway and heat cytotoxicity. First, we performed immuofluorescent staining of endogenous Rad9 with anti-Rad9 antibody to analyze its subnuclear localization during heat stress. When HeLa cells, transfected with siRNA against *GFP* (as negative control), were pre-extracted by Triton X-100 before fixing with paraformaldehyde, Rad9 signal was detected and visualized as subnuclear foci, whose intensity reduced significantly by siRNA-mediated knockdown of *Rad9* (Fig. S1A). This result indicates that this anti-Rad9 antibody specifically reacted with endogenous Rad9, which accumulates in detergent-resistant subnuclear fraction, possibly chromatin fraction, in normal culture condition. When HeLa cells were incubated at 42.5°C for 30 minutes, similar subnuclear foci of Rad9 were detected, while RPA32 subnuclear foci were not detected (Fig. S1B). In contrast, when cells were treated with 5 mM HU for 3 hours, subnuclear foci of Rad9 were also detected, but some cells were positively stained with RPA32 (Fig. S1B, indicated by white arrowheads). Collectively, these results suggest that Rad9 resided in chromatin fraction even though RPA32 was not actively accumulated in chromatin fraction when cells were exposed to heat stress.

**Figure 2:**
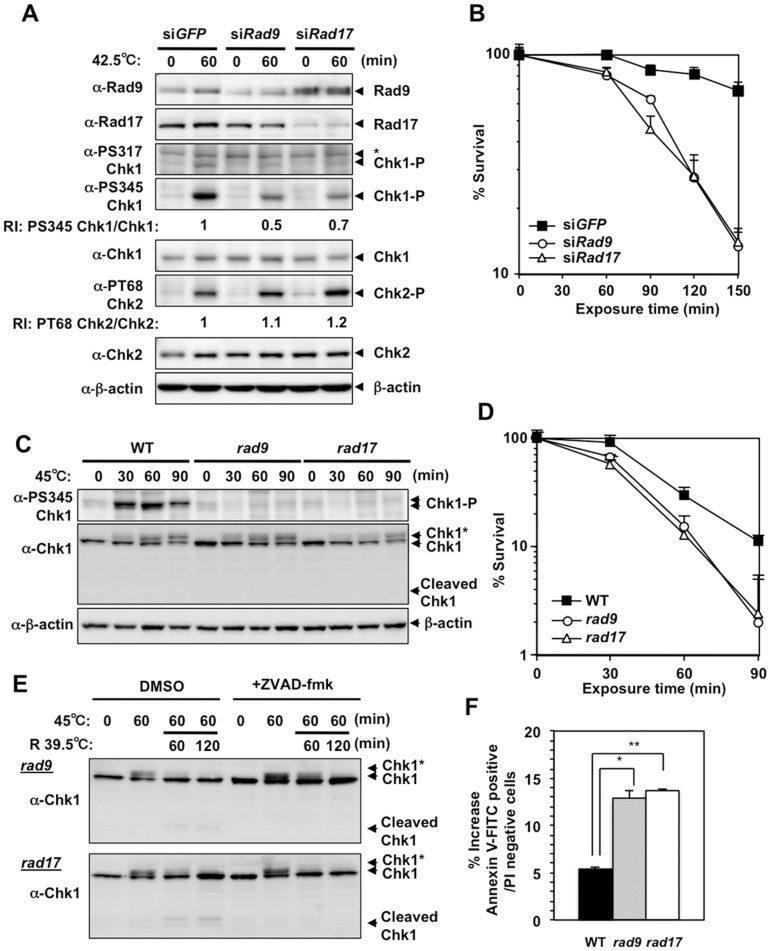
Rad9- or Rad17-deficiency inhibited heat-induced Chk1 phosphorylation at Ser345 and enhanced heat cytotoxicity. **A.** Western blot. HeLa cells were transfected with siRNA for *GFP, Rad9* or *Rad17* and cultured at 42.5°C for 60 minutes. Non-specific bands were indicated as *. RI: relative intensity compared to the sample of si*GFP* and 42.5°C for 60 minutes. **B.** Clonogenic survival. HeLa cells were transfected with siRNA for *GFP, Rad9* or *Rad17* and cultured at 42.5°C for the indicated time. **C.** Western blot. Wild-type, *Rad9*- and *Rad17-*deficient DT40 cells (WT, *rad9* or *rad17*) were cultured at 45°C for the indicated time. **D.** Clonogenic survival. WT, *rad9* and *rad17* DT40 cells were cultured at 45°C for the indicated time. **E.** Western blot. The *rad9* and *rad17* DT40 cells were cultured at 45°C for 60 minutes and at 39.5°C for the indicated time in the presence of DMSO or caspase inhibitor (50 µM ZVAD-fmk). **F.** The induction of early apoptotic cells by heat. Early apoptotic cells were detected as annexin V-FITC-positive, propidium iodide (PI)-negative population. WT, *rad9* and *rad17* DT40 cells were cultured at 45°C for 60 minutes and at 39.5°C for 60 minutes, and the increase in early apoptotic cells induced by these treatment is shown. *p = 0.0016, **p = 0.0002 (Student's *t* test).

When HeLa cells were treated with siRNA targeting *Rad9* or *Rad17*, heat-induced Chk1 Ser317 and Ser345 phosphorylation was suppressed, while heat-induced Chk2 Thr68 phosphorylation was slightly increased (Fig. 2A). SiRNA-mediated knockdown of *Rad9* or *Rad17* in HeLa cells reduced clonogenic viability at the higher temperature (Fig. 2B). When *Rad9*- or *Rad17*-deficient DT40 cells (*rad9* or *rad17*) [21] were incubated at 45°C, Ser345 phosphorylation of Chk1 was hardly detectable (Fig. 2C). The *rad9* or *rad17* cells also exhibited reduced clonogenic viability at the higher temperature (Fig. 2D). In addition, the cleaved Chk1 peptide was clearly detected when these cells were shifted to 39.5°C after a 1-hour incubation at 45°C (Fig. 2E), while that peptide was hardly detectable when wild-type cells were treated similarly (Fig. S3E). Because this peptide was not detected in the presence of the caspase inhibitor, ZVAD-fmk (Fig. 2E), the peptide must have been produced by caspase-mediated cleavage during apoptosis induced at 45°C [22], [23]. Chk1 peptide produced by caspase-mediated cleavage at Asp299 was detected when cells undergo apoptosis and a truncated form of Chk1 mimicking the N-terminal cleavage fragment (residue 1–299) is implicated in enhancing apoptotic reactions [22]. Consistently, the increase in annexin V-positive, PI-negative population was more prominent in heat-treated *rad9* and *rad17* cells than in heat-treated wild-type cells (Fig. 2F). These results indicate that Rad9 and Rad17 were required for activation of the ATR-Chk1 pathway by heat and were involved in the suppression of heat-induced apoptosis, and contributed to the increase in clonogenic viability. Of note, slower migrating forms of Chk1 (Chk1*) were detected in *rad9* and *rad17* cells, suggesting that this posttranslational modification of Chk1 still occurred in the absence of Rad9- or Rad17-dependent ATR activation.

### siRNA-mediated knockdown of TopBP1 and Claspin suppressed heat-induced Chk1 Ser345 phosphorylation and enhanced heat cytotoxicity

In the activation of ATR-Chk1 pathway during stalled replication forks, Rad9 and Rad17 cooperate with several essential factors, such as TopBP1 and Claspin [15]. Endogenous TopBP1 was positively stained with anti-TopBP1 antibody by immunofluorescence in detergent pre-extracted HeLa cells, whose intensity decreased significantly by siRNA-mediated knockdown of *TopBP1* (Fig. S1C), confirming the specificity of anti-TopBP1 antibody and its chromatin localization. When HeLa cells were cultured at 42.5°C for 30 min, the detergent-resistant immunofluorescence signal of TopBP1 was similarly detected, while that of RPA32 was not (Fig. S1D). When HeLa cells were cultured in the presence of 5 mM HU for 3 hours (Fig. S1D), the detergent-resistant immunofluorescence signal of TopBP1 was detected, but in this case, some cells were also positively immunostained with RPA32 (Fig. S1D). These results suggest that TopBP1 resided in the chromatin fraction even though RPA32 was not actively accumulated in chromatin fraction when cells were exposed to heat stress.

**Figure 3:**
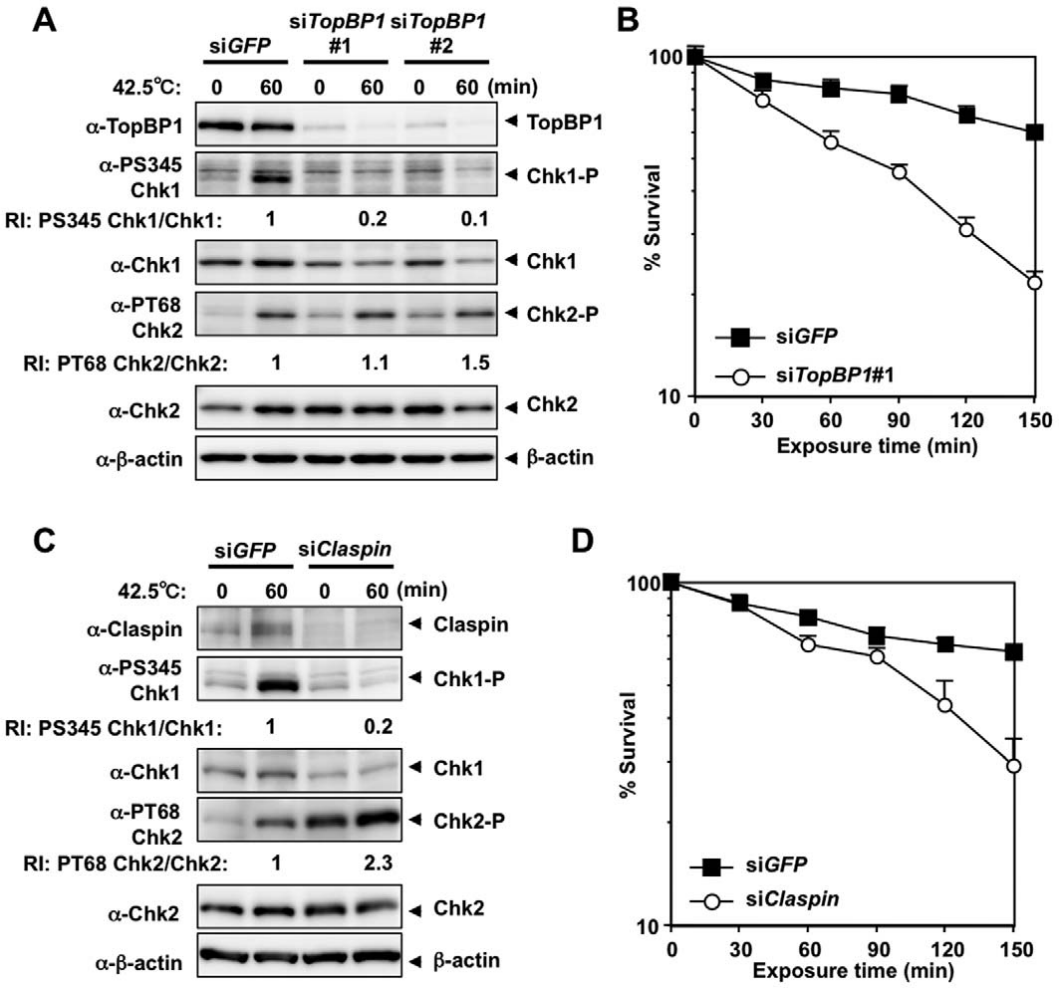
siRNA-mediated knockdown of TopBP1 and Claspin suppressed heat-induced Chk1 Ser345 phosphorylation and enhanced heat cytotoxicity. **A.** Western blot. HeLa cells were transfected with siRNA for *GFP, TopBP1*#1 or *TopBP1#2* and cultured at 42.5°C for 60 minutes. RI: relative intensity compared to the sample of si*GFP* and 42.5°C for 60 minutes. **B.** Clonogenic survival. HeLa cells were transfected with siRNA for *GFP or TopBP1*#1 and cultured at 42.5°C for the indicated time. **C.** Western blot. HeLa cells were transfected with siRNA for *GFP* or *Claspin* and cultured at 42.5°C for 60 minutes. RI: relative intensity compared to the sample of si*GFP* and 42.5°C for 60 minutes. **D.** Clonogenic survival. HeLa cells were transfected with siRNA for *GFP or Claspin* and cultured at 42.5°C for the indicated time.

To test whether TopBP1 and Claspin are also involved in the activation of ATR-Chk1 pathway by heat or heat tolerance, we knocked down *TopBP1* or *Claspin* by siRNA in HeLa cells and analyzed heat-induced phosphorylation of Chk1 and Chk2 or heat cytotoxicity by measuring clonogenic viability. Heat-induced Chk1 Ser345 phosphorylation was significantly suppressed by siRNA-mediated knockdown of TopBP1 (Fig. 3A) or Claspin (Fig. 3C), while heat-induced Chk2 Thr68 phosphorylation was slightly enhanced (Fig. 3A and 3C). Furthermore, siRNA-mediated knockdown of *TopBP1* (Fig. 3B) or *Claspin* (Fig. 3D) decreased clonogenic viability to heat stress significantly. These results indicate that TopBP1 and Claspin were also required for the activation of ATR-Chk1 pathway by heat stress and contributed to the increase in clonogenic viability.

### ATM-deficiency results in mild heat sensitivity that is independent of ATR kinase activity

**Figure 4:**
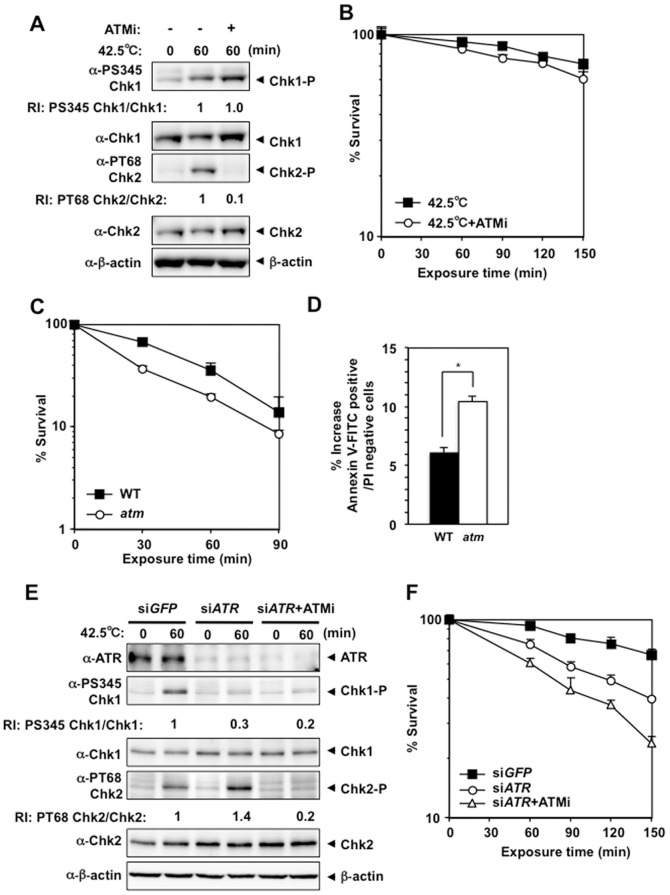
ATM-deficiency inhibited heat-induced Chk2 Thr68 phosphorylation and enhanced heat cytotoxicity. **A.** Western blot. HeLa cells were cultured at 42.5°C for 60 minutes in the presence or absence of the ATM inhibitor KU55933 (ATMi). RI: relative intensity compared to the sample of ATMi (−) and 42.5°C for 60 minutes. **B.** Clonogenic survival. HeLa cells were cultured at 42.5°C for the indicated time in the presence or absence of ATMi. **C.** Clonogenic survival. Wild-type and *Atm-*deficient DT40 cells (WT or *atm*) were cultured at 45°C for the indicated time. **D.** Apoptosis. WT and *atm* DT40 cells were cultured at 45°C for 60 minutes and at 39.5°C for 60 minutes, and the increase in the number of early apoptotic cells induced by these treatments is shown. *p = 0.0131 (Student's *t* test). **E.** Western blot. HeLa cells transfected with siRNA for *GFP* or *ATR* (in the presence or absence of ATMi) were cultured at 42.5°C for 60 minutes. RI: relative intensity compared to the sample of si*GFP* and 42.5°C for 60 minutes. **F.** Clonogenic survival. HeLa cells transfected with siRNA for *GFP* or *ATR* (in the presence or absence of ATMi) were cultured at 42.5°C for the indicated time.

Next, we examined the possible involvement of ATM kinase activity in heat tolerance. In the presence of ATM inhibitor, KU55933, heat-induced Chk2 Thr68 phosphorylation was significantly suppressed, while Chk1 Ser345 phosphorylation was normally induced (Fig. 4A). Clonogenic viability at the higher temperature decreased only slightly in the presence of KU55933 (Fig. 4B). *ATM*-deficient DT40 cells (*atm*) also exhibited slight heat sensitivity (Fig. 4C), while heat-induced Ser345 phosphorylation and slower migrating forms of Chk1 (Chk1*) were detected at normal levels (Fig. S2A). Cleaved Chk1 peptide, which was also suppressed by ZVAD-fmk, was detected when cells were shifted to 39.5°C after a 1-hour incubation at 45°C (Fig. S2B), and the increase in annexin V-positive, PI-negative population was more prominent in heat-treated *atm* cells than in heat-treated wild-type cells (Fig. 4D). To determine whether the ATR-Chk1 and ATM-Chk2 pathways contribute to heat tolerance in a non-overlapping manner, we analyzed cellular responses and clonogenic viability at the higher temperature in KU55933-treated HeLa cells treated with *ATR* siRNA. SiRNA knockdown of ATR suppressed heat-induced Chk1 Ser345 phosphorylation and slightly enhanced heat-induced Chk2 Thr68 phosphorylation (Fig. 4E), and reduced the clonogenic viability of HeLa cells at the higher temperature (Fig. 4F). KU55933 suppressed the increased phosphorylation of Chk2 Thr68 (Fig. 4E) and increased the heat sensitivity of HeLa cells treated with *ATR* siRNA (Fig. 4F). This result clearly supports the idea that the ATR-Chk1 and ATM-Chk2 pathways contribute to heat tolerance in a non-overlapping manner.

### Caffeine suppressed heat-induced phosphorylation of Chk1 Ser345 and Chk2 Thr68 and enhanced heat cytotoxicity

**Figure 5:**
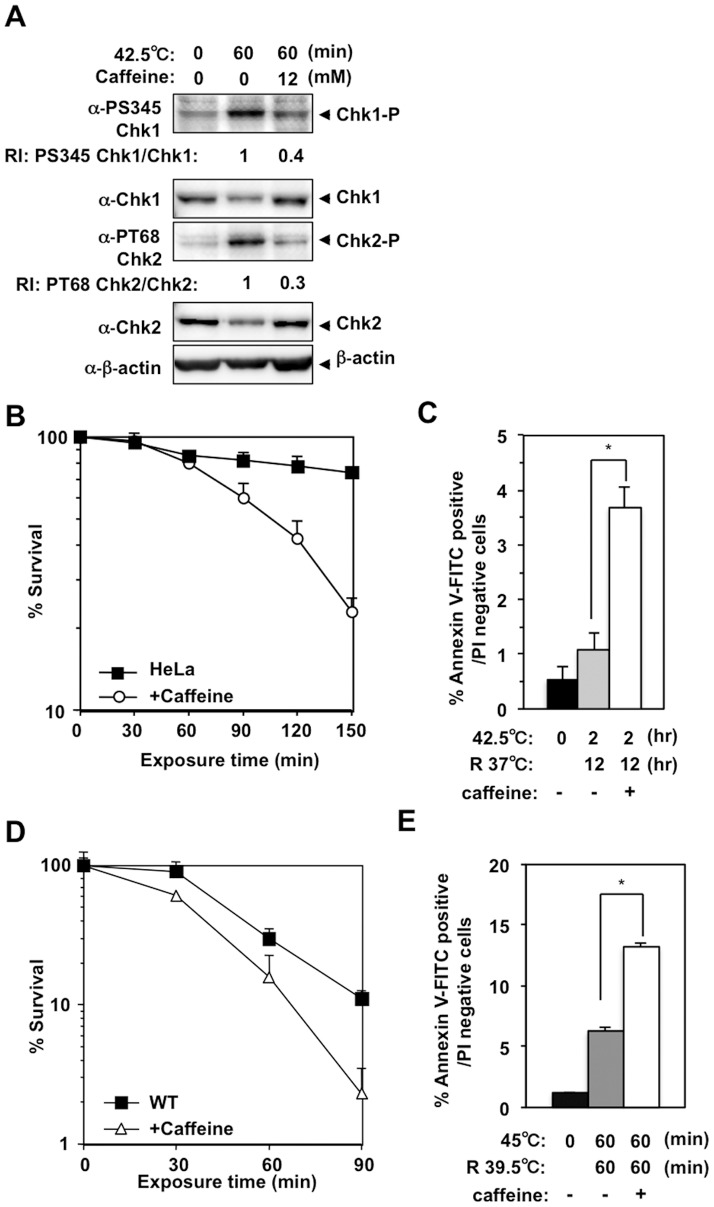
Caffeine suppressed heat-induced phosphorylation at Chk1 Ser345 and Chk2 Thr68 and enhanced heat cytotoxicity. **A.** Western blot. HeLa cells were cultured at 42.5°C for 1 hour in the presence or absence of 12 mM caffeine. RI: relative intensity compared to the sample of 42.5°C for 60 minutes. **B.** Clonogenic survival of heat-treated HeLa cells in the presence or absence of 12 mM caffeine. **C.** Apoptosis. HeLa cells were cultured at 42.5°C for 2 hours and at 37°C for 12 hours in the presence or absence of 12 mM caffeine. *p = 0.0019 (Student's *t* test). **D.** Clonogenic survival of WT DT40 cells cultured at 45°C for the indicated time in the presence or absence of 2 mM caffeine. **E.** Apoptosis. WT DT40 cells were cultured at 45°C for 60 minutes and at 39.5°C for 60 minutes in the presence or absence of 2 mM caffeine. *p = 0.0014 (Student's *t* test).

Caffeine is an inhibitor of both ATM and ATR kinase activity [24]. We examined whether caffeine had any effect on heat-induced phosphorylation of Chk1 Ser345 and Chk2 Thr68, and on heat cytotoxicity. In HeLa cells, heat-induced phosphorylation of Chk1 Ser345 and Chk2 Thr68 was significantly suppressed when 12 mM caffeine was added to the medium (Fig. 5A). Clonogenic viability also decreased significantly at the higher temperature in the presence of caffeine (Fig. 5B). Consistently, cells in annexin V-positive, PI-negative population (Fig. 5C) and in subG1 population (Fig. S3A) increased significantly in the presence of caffeine when cells were shifted to 37°C after a 2-hour incubation at 42.5°C. Similarly, in DT40 cells, 2 mM caffeine suppressed heat-induced Chk1 Ser345 phosphorylation (Fig. S3B) and significantly decreased clonogenic viability (Fig, 5D). These effects were observed more clearly as the concentration of caffeine increased (Fig. S3C and S3D). In the presence of 2 mM caffeine, the cleaved Chk1 peptide was detected when cells were shifted to 39.5°C after a 1-hour incubation at 45°C (Fig. S3E). Cells in annexin V-positive, PI-negative population (Fig. 5E) and in subG1 population (Fig. S3F) increased significantly in the presence of caffeine. Even though we were not able to evaluate caffeine's effect on heat-induced Chk2 phosphorylation in DT40 cells due to unavailability of appropriate antibodies, these results suggest that caffeine may have enhanced heat cytotoxicity by suppressing both ATM and ATR kinase activities. Of note, slower migrating forms of Chk1 were still normally detected even in the presence of 8 mM caffeine, while phosphorylation at Ser345 was nearly completely abolished (Fig. S3D). This result also suggests that this posttranslational modification of Chk1 was not dependent on ATM/ATR kinase activity.

### Caffeine enhanced the heat cytotoxicity of rad9, rad17, and atm cells by increasing apoptosis

**Figure 6:**
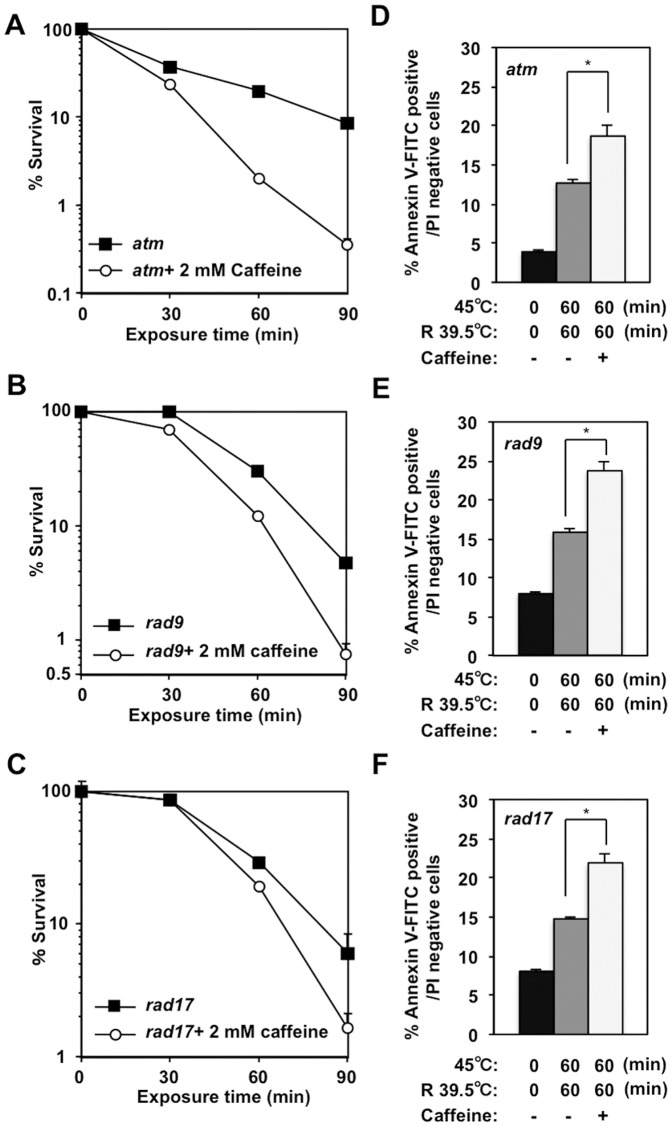
Caffeine enhanced the heat cytotoxicity of *rad9, rad17* or *atm* DT40 cells. **A–C.** Clonogenic survival. *atm* (**A**), *rad9* (**B**) and *rad17* (**C**) DT40 cells were cultured at 45°C for the indicated time in the presence or absence of 2 mM caffeine. **D–F.** Apoptosis. *atm* (**D**), *rad9* (**E**) and *rad17* (**F**) DT40 cells were cultured at 45°C for 60 minutes and at 39.5°C for 60 minutes in the presence or absence of 2 mM caffeine. (**D**) *p = 0.0012, (**E**) *p = 0.0057, (**F**) *p = 0.0084 (Student's *t* test).

To identify the principal target of caffeine in heat cytotoxicity, we performed a clonogenic survival assays for *rad9, rad17* and *atm* cells in the presence of 2 mM caffeine. When 2 mM caffeine was added to *atm* cells during heat treatment, heat-induced Chk1 Ser345 phosphorylation was suppressed (Fig. S4A) and clonogenic viability was decreased (Fig. 6A). Clonogenic viability of *rad9* (Fig. 6B) and *rad17* (Fig. 6C) cells was also decreased when these mutant cells were cultured at high temperature in the presence of 2 mM caffeine. A slight increase in the amount of the caspase-cleaved Chk1 peptide was detected when caffeine was added to heat-treated *rad9* (Fig. S4C), *rad17* (Fig. S4D) and *atm* cells (Fig. S4B). Consistently, caffeine induced an increase in the number of cells in annexin V-positive, PI-negative population among *rad9* (Fig. 6E), *rad17* (Fig. 6F) and *atm* (Fig. 6D) cells shifted to 39.5°C after a 1-hour incubation at 45°C. These data further support the idea that heat-induced activation of both ATM and ATR kinases contributes to heat tolerance and that caffeine enhances heat cytotoxicity by inhibiting both ATM and ATR kinases.

## Discussion

Hyperthermia exerts pleiotropic effects on proliferating cells and causes cytotoxicity. From the analysis of cellular responses to hyperthermia, we found that the ATR-Chk1 pathway contributes to heat tolerance and that Rad9, Rad17, TopBP1 and Claspin are absolutely required for activation of the ATR-Chk1 pathway at high temperature. ATM-Chk2 pathway was also activated by heat and contributed to heat tolerance mildly but significantly. The ATR-Chk1 and ATM-Chk2 pathways contributed to heat tolerance in a non-overlapping manner and simultaneous inhibition of ATR and ATM kinases significantly enhanced cytotoxicity to hyperthermia.

Rad9 and Rad17 were important for heat-induced activation of the ATR-Chk1 pathway and for heat tolerance (Fig. 2). Rad9 is a component of the 9-1-1 heterotrimeric clamp that binds to 5′ ends of the primer-template junctions containing exposed regions of ssDNA, and Rad17 is an essential component of the 9-1-1-clamp loader complex. Both of these factors are required for activation of the ATR-Chk1 pathway, particularly when replication forks are stalled [20]. The involvement of Rad9 and Rad17 in the heat response suggests that ssDNA and 5′ ends of primer-template junctions are generated during hyperthermia. This idea is supported by our previous study using the *in situ* nick translation method, which revealed the presence of DNA strand scissions in HeLa cells upon exposure to heat [6]. Such DNA structures might be formed when DNA synthesis ceases incompletely during replication process. Furthermore, we also found that heat-induced Chk1 Ser345 phosphorylation was significantly suppressed by siRNA-mediated downregulation of TopBP1 (Fig. 3A), which plays an essential role in the activation of ATR kinase via its activation domain through direct binding to phosphorylated Rad9 at damaged DNA [25]. SiRNA-mediated downregulation of Claspin, which is an essential upstream regulator of Chk1 [26], also suppressed heat-induced Chk1 Ser345 phosphorylation (Fig. 3C). These results strongly indicate that common mechanism is involved in heat-induced activation of the ATR-Chk1 pathway.

Heat-induced activation of the ATR-Chk1 pathway was not associated with FancD2 monoubiquitination, RPA32 phosphorylation or chromatin accumulation of RPA70/RPA32 (Fig. 1E). This is quite different from cellular responses induced by HU or DNA crosslinkers, which causes DNA damage associated with stalled replication forks. During activation of the ATR-Chk1 pathway by DNA damage (IR) or stalled replication forks (HU, ultraviolet irradiation), ssDNA is coated by the trimeric RPA complex, which recruits ATR-ATRIP complex to sites of DNA damage [27]. For the induction of FancD2 monoubiquitination, in addition to functional FA core complex [19], ATR-mediated phosphorylation of FancI [17] and ATRIP binding to RPA70 [28], are required. However, a previous report showed that RPA32 nuclear foci do not form during hyperthermia [13]. We ourselves confirmed this result (Fig. S1B and S1D). The recruitment of RPA32 to ssDNA might be inhibited through its direct sequestering by nucleolin, which relocalizes from the nucleolus to nucleoplasm and increases its binding to RPA32 by heat stress [29]. Even though ATRIP is supposed to recognize RPA-ssDNA complex to sense DNA damage [27], other report shows that RPA32 downregulation do not suppress HU-induced Chk1 Ser345 phosphorylation [30]. In addition, Chk1 Ser345 phosphorylation occurs in the absence of RPA32 through the direct binding of ATRIP to DNA in *Xenopus* system [31]. The activation of ATR kinase and phosphorylation of Chk1 Ser345 could occur in the absence of functional RPA-ssDNA complex at damage site during hyperthermia, but the downstream events, such as RPA32 phosphorylation or FancD2 monoubiquitination, might be perturbed because of its absence.

The heat-induced emergence of slow migrating forms of Chk1 in DT40 cells (Fig. 1B) indicated that heat induced posttranslational modification(s) of Chk1. The slow migrating forms of Chk1 were also detected even in heat-treated *rad9*, *rad17* (Fig. 2C) and *atm* cells (Fig. S2A). These forms were still detectable even in caffeine-treated wild type (Fig. S3B), *rad9* (Fig. S4C), *rad17* (Fig. S4D) and *atm* cells (Fig. S4B). This result suggests that such posttranslational modifications of Chk1 occur in ATM- and ATR-independent manner. This modification may alter Chk1 function or activity. We are currently interested in this possibility and trying to clarify its possible role in cellular response to heat and heat tolerance.

**Figure 7:**
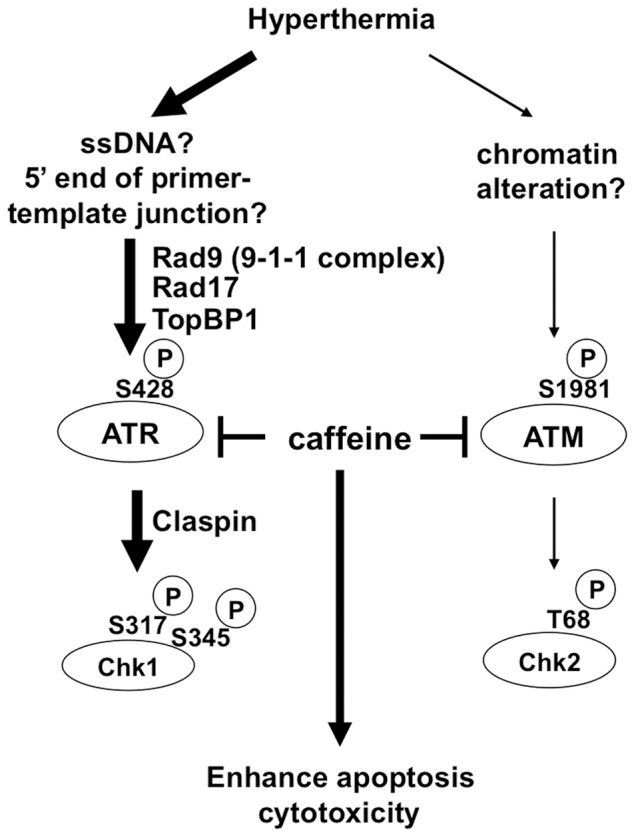
Model of cellular response to heat stress. See text for details.

Both the ATR-Chk1 and ATM-Chk2 pathways were activated by heat and contributed to heat tolerance in a non-overlapping manner (Fig. 7). Consistent with a previous report [13], ATR was preferentially activated by heat and contributed more to heat tolerance than ATM. Furthermore, Rad9, Rad17, TopBP1 and Claspin were required for heat-induced ATR activation and heat tolerance. Interestingly, not all downstream pathways of ATR kinase were activated by heat treatment, indicating that ATR activation by hyperthermia has distinct biological consequences. Finally, inhibition of ATM and ATR kinase activity at the same time by caffeine was effective way to enhance heat cytotoxicity, which could have clinical implication. The activation of DNA damage signaling by heat may compromise normal DNA damage responses. Our findings may provide some clues to understand why hyperthermia potentiates the cytotoxic effects of radiation therapy and chemotherapy and help us to improve hyperthermia therapeutic strategy.

## Materials and Methods

### Cell lines, cell culture and reagents

HeLa cells were cultured at 37°C in DMEM supplemented with 10% FBS. The chicken B lymphoma cell line DT40 and its mutants (*rad9* [21], *rad17* [21] or *atm* [32]) were cultured at 39.5°C in RPMI1640 supplemented with 10% fetal bovine serum (FBS), 1% chicken serum, penicillin-streptomycin, L-glutamine and β-mercaptoethanol, as described previously [33]. UCN-01 and KU55933 were purchased from Sigma, caffeine was from Nacalai Tesque, and caspase inhibitor ZVAD-fmk was from MBL.

### siRNA transfection

The following siRNAs were used: *Rad9:* 5′-GCAAACUUGAAUCUUAGCA-3′; *Rad17:* 5′-CAAGUACAAGAGUGGAUUA-3′; *ATR:* 5′-CCUCCGUGAUGUUGCUUGA-3′ [34]. *TopBP1*#1: 5′-CUCACCUUAUUGCAGGAGA-3′; *TopBP1*#2: 5′-CUCACCUUAUUGCAGGAGA-3′ [35]; *Claspin:* 5′-GCACAUACAUGAUAAAGAA-3′, *GFP:* 5′-UCUUAAUCGCGUAUAAGGC-3′. siRNAs were transfected using RNAiMax (Invitrogen).

### Clonogenic survival assay

Clonogenic survival assay was performed with DT40 cells as described previously [36] with the following modifications. Briefly, 1×10^4^ cells were suspended in 1 ml culture media with or without caffeine in an eppendorf tube. After 10 minutes preincubation at 39.5°C, the cells were exposed to heat by placing each tube in a water bath at 45°C. After incubation for the indicated times, 1×10^2^ cells were plated on methylcellulose-containing media, and incubated for 1–2 weeks at 39.5°C. Emerging colonies were counted. For the HeLa cell, 2×10^2^ cells were inoculated into 60 mm^2^ plates and incubated at 37°C for 24 hours. Cells were exposed to 42.5°C for the indicated times and incubated at 37°C for 10 days. Emerging colonies were stained with crystal violet and counted. All experiments were done in triplicate.

### Western blot analysis

For DT40 cells, 5×10^5^ cells were suspended in 1 ml culture media in an eppendorf tube and incubated at 45°C in water bath. After incubation for the indicated times, cells were collected by centrifugation and re-suspended in 1×SDS sample buffer. For HeLa cells, 5×10^5^ cells were incubated at 42.5°C for the indicated times and harvested. Collected cells were lysed in RIPA buffer (1.0% NP40; 50 mM Tris HCl, pH 8.0; 150 mM NaCl; 0.5% deoxycholate; 0.1% SDS; 2 mM phenylmethylsulfonyl fluoride (PMSF); 2 mM NaF and 2 mM Na_3_VO_4_ with protease inhibitor cocktail (Nacalai Tesque)) for 30 minutes at 4°C. The protein concentration of extracts and cleared lysates were determined by the RC DC Protein Assay Kit (Bio-Rad). Equal amounts of protein (10 µg/lane) were subjected to SDS-PAGE. The following antibodies were used; Anti-chicken FancD2 (kindly provided by Prof. Komatsu, Radiation Biology Center, Kyoto University), anti-Chk1 (G4, Santa Cruz), anti-Phospho-Chk1 (Ser345) (#2341, Cell Signaling), anti-Chk2 (1C12) (#3440, Cell Signaling), anti-Phospho-Chk2 (Thr68) (#2661, Cell Signaling), anti-Rad9 (M-389, Santa Cruz), anti-ATR (#2790, Cell Signaling), anti-Rad17 (H-300, Santa Cruz), anti-β-actin (AC-74, Sigma), anti-TopBP1 (AB3245, Millipore), anti-RPA70 (#2589-1, Epitomics), anti-RPA32 (#2461-1, Epitomics), anti-FancD2 (LS-B493, LS Bio), anti-Claspin (A300-266A, Bethyl) and anti-histone H3 (H9289, Sigma). Relative intensity of phosphorylation level of Chk1 (Ser345) and Chk2 (Thr68) were determined by band intensity measured by Image J software (NIH).

### Cell cycle analysis

Cells were exposed to heat for the indicated times and fixed with 70% ethanol immediately. DNA contents were analyzed using fixed cells treated with propidium iodide (PI) and RNaseA. The samples were analyzed using FACSCalibur (BD Biosciences) and % of subG1 population (<2N) was calculated.

### Detection of early apoptotic cells using Annexin V-FITC

Early apoptotic cells were detected using an Annexin V–FITC apoptosis detection kit (Sigma) as described previously [23]. Briefly, 5×10^5^ cells were resuspended in 0.5 ml of 1× binding buffer (10 mM HEPES/NaOH, pH 7.5, 140 mM NaCl, 2.5 mM CaCl_2_) and stained with 0.5 µg/ml of the annexin V–FITC conjugate and 2 µg/ml PI for 10 minutes at room temperature before FACS analysis. Annexin V–FITC-positive, PI-negative cells were counted as early apoptotic cells. Experiments were done in triplicate.

### Isolation of chromatin plus nuclear matrix fraction from HeLa cells

Subcellular fractionation was done as described previously [37]. Briefly, cells were resuspended (4×10^7^ cells/ml) in buffer A (10 mM HEPES [pH 7.9], 10 mM KCl, 1.5 mM MgCl_2_, 0.34 M Sucrose, 10% Glycerol, 1 mM DTT, 0.1 mM PMSF with protease inhibitor cocktail). 0.1% Triton X-100 was added and the cells were incubated on ice for 5 minutes. Nuclei were collected in pellet by low-speed centrifugation (4 minutes, 1,300× *g*, 4°C). Nuclei were washed once in buffer A, and then lysed in buffer B (3 mM EDTA, 0.2 mM EGTA, 1 mM DTT, 0.1 mM PMSF with protease inhibitor cocktail). Insoluble chromatin plus nuclear matrix fraction was collected in pellet by centrifugation (4 minutes, 1,700× *g*, 4°C) and washed once in buffer B. Final pellet was resuspended in 1xSDS buffer and sonicated. Protein concentration was determined by the RC DC Protein Assay Kit and appropriate amount of protein was subjected to SDS-PAGE and Western blot.

### Fluorescence immunostaining

Immunostaining was done as described previously [38]. Cells were grown as monolayers on glass coverslips and pre-extracted in Buffer 1 (1% Triton X-100, 10 mM HEPES pH 7.4, 10 mM NaCl, 3 mM MgCl_2_) for 5 minutes at 4°C. Cells were then fixed in 4% paraformaldehyde for 10 minutes at 4°C and permeabilized further in Buffer 2 (0.5% Triton X-100, 20 mM HEPES pH 7.4, 50 mM NaCl, 3 mM MgCl_2_, 300 mM sucrose) for 5 minutes at 4°C. To detect endogenous Rad9, RPA32 and TopBP1, the following primary antibodies were used; Rad9 (IHC-00376, Bethyl, 1∶50 dilution), RPA32 (GTX16855, Genetex, 1∶50 dilution) and TopBP1 (AB3245, Millipore, 1∶100 dilution). Cells were stained with an Alexa488- or Alexa594-conjugated secondary antibody (Invitrogen) and the nuclei were counterstained with 4′,6-diamino-2-phenylindole (DAPI). All images were captured using a BIOREVO BZ-9000 fluorescence microscope (Keyence).

## Supporting Information

Figure S1 **Chromatin localization of Rad9 and TopBP1 in HeLa cells.** **A.** Immunofluorescence staining with anti-Rad9 antibody. HeLa cells were treated with siRNA of *GFP* or *Rad9*, pre-extracted by detergent and immunostained with anti-Rad9 antibody. Nuclei were counterstained with 4′,6-diamino-2-phenylindole (DAPI). White arrowhead indicates a cell in M phase. **B.** Coimmunostaining of Rad9 and RPA32. HeLa cells were cultured at 37°C, 42.5°C or in the presence of 5 mM hydroxyurea (HU), pre-extracted by detergent and coimmunostained with Rad9 and RPA32 antibodies. Nuclei were counterstained with DAPI. White arrowheads indicate RPA32-positive cells. **C.** Immunofluorescence staining with anti-TopBP1 antibody. HeLa cells were treated with siRNA of *GFP* or *TopBP1*, pre-extracted by detergent and immunostained with anti-TopBP1 antibody. Nuclei were counterstained with DAPI. **D.** Coimmunostaining of TopBP1 and RPA32. White arrowheads indicate RPA32-positive cells.
(TIFF)

Figure S2 **Cellular response to heat in** ***ATM-*** **deficient DT40 cells.** **A.** Western blot. Wild-type (WT) and *ATM-*deficient (*atm*) DT40 cells were cultured at 45°C for the indicated time. RI: relative intensity compared to the sample of 45°C for 15 minutes in WT DT40 cells. **B.** Disappearance of the cleaved Chk1 peptide following treatment with the caspase inhibitor, ZVAD-fmk. *atm* cells were cultured at 45°C for 60 minutes and at 39.5°C for the indicated time in the presence or absence of 50 µM ZVAD-fmk.
(TIFF)

Figure S3 **Caffeine enhanced heat cytotoxicity.** **A.** SubG1 population. HeLa cells were cultured at 42.5°C for 2 hours and at 37°C for 12 or 24 hours in the presence or absence of 12 mM caffeine. *p = 0.0016, **p = 0.0002 (Student's *t* test). **B.** Western blot. Wild-type DT40 cells (WT) were cultured at 45°C for the indicated time in the presence or absence of 2 mM caffeine. **C.** Clonogenic survival. WT DT40 cells were cultured at 45°C for the indicated time in the presence of various concentration of caffeine. **D.** Western blot. WT DT40 cells were cultured at 45°C for 30 minutes in the presence of various concentration of caffeine. RI: relative intensity compared to the sample of 45°C for 30 minutes without caffeine in WT DT40 cells. **E.** Western blot. WT DT40 cells were cultured at 45°C for 60 minutes and at 39.5°C for the indicated time in the presence or absence of 2 mM caffeine. **F.** SubG1 population. WT DT40 cells were cultured at 45°C for 60 minutes and at 39.5°C for 60 minutes in the presence or absence of 2 mM caffeine. *p = 0.0069 (Student's *t* test).
(TIFF)

Figure S4 **Cellular response to heat in the presence of caffeine in mutant DT40 cells.** **A.** Western blot. *ATM-*deficient DT40 cells (*atm*) were cultured at 45°C for the indicated time in the presence or absence of 2 mM caffeine. **B–D.** Western blot. *atm* (**B**), *rad9* (**C**) and *rad17* (**D**) DT40 cells were cultured at 45°C for 60 minutes and at 39.5°C for the indicated time in the presence or absence of 2 mM caffeine. The percentage of cleaved Chk1 peptide per total.
(TIFF)
