# Supplementary figures and images for: Rad9, Rad17, TopBP1 and Claspin Play Essential Roles in Heat-Induced Activation of ATR Kinase and Heat Tolerance
Source: PLoS One. 2013 Feb 1;8(2):e55361. doi: 10.1371/journal.pone.0055361 (PMC3562228; doi:10.1371/journal.pone.0055361)

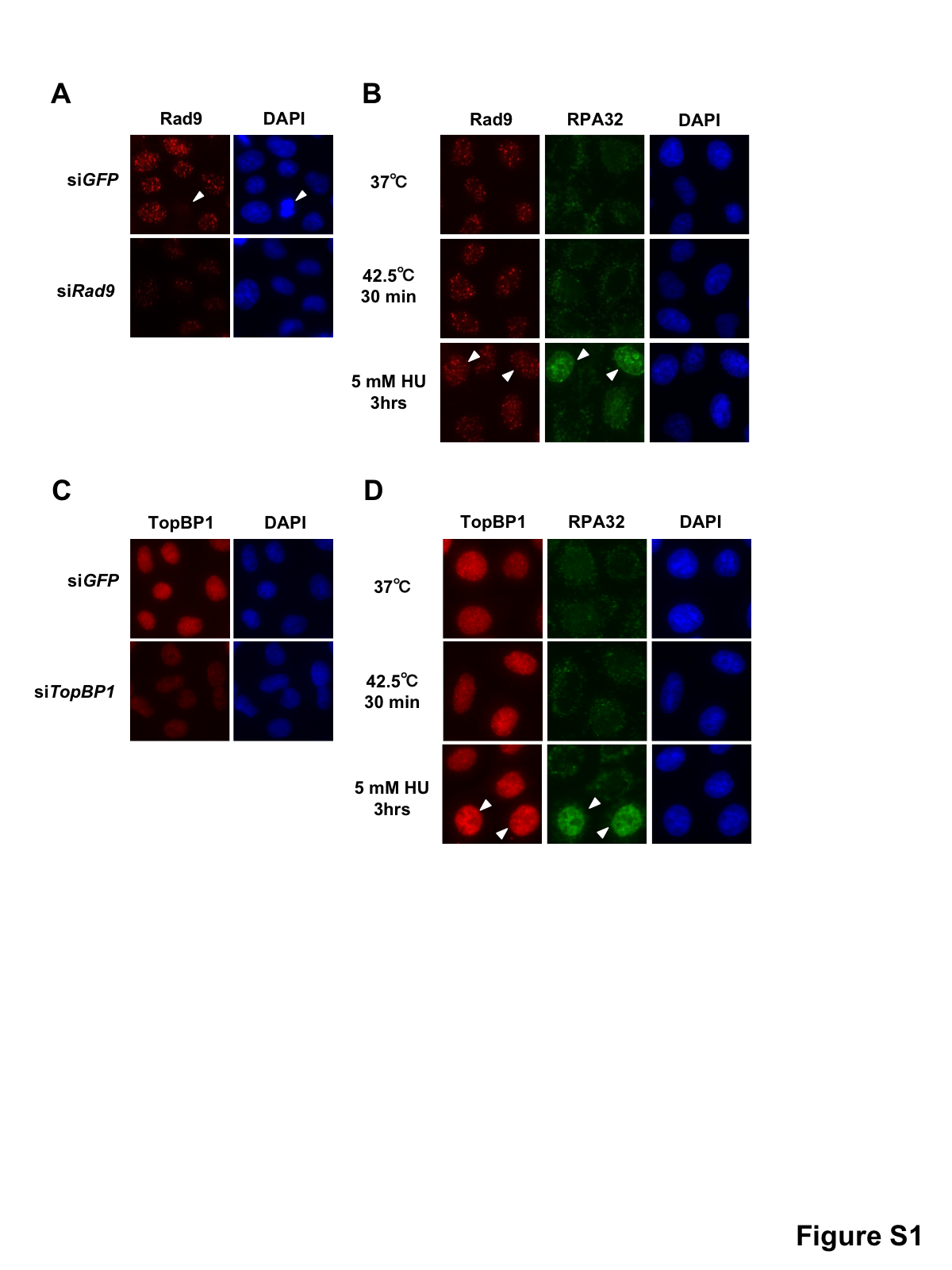

Supplement: Figure S1 — Chromatin localization of Rad9 and TopBP1 in HeLa cells. A. Immunofluorescence staining with anti-Rad9 antibody. HeLa cells were treated with siRNA of GFP or Rad9, pre-extracted by detergent and immunostained with anti-Rad9 antibody. Nuclei were counterstained with 4′,6-diamino-2-phenylindole (DAPI). White arrowhead indicates a cell in M phase. B. Coimmunostaining of Rad9 and RPA32. HeLa cells were cultured at 37°C, 42.5°C or in the presence of 5 mM hydroxyurea (HU), pre-extracted by detergent and coimmunostained with Rad9 and RPA32 antibodies. Nuclei were counterstained with DAPI. White arrowheads indicate RPA32-positive cells. C. Immunofluorescence staining with anti-TopBP1 antibody. HeLa cells were treated with siRNA of GFP or TopBP1, pre-extracted by detergent and immunostained with anti-TopBP1 antibody. Nuclei were counterstained with DAPI. D. Coimmunostaining of TopBP1 and RPA32. White arrowheads indicate RPA32-positive cells. (TIFF) [file pone.0055361.s001.tiff]

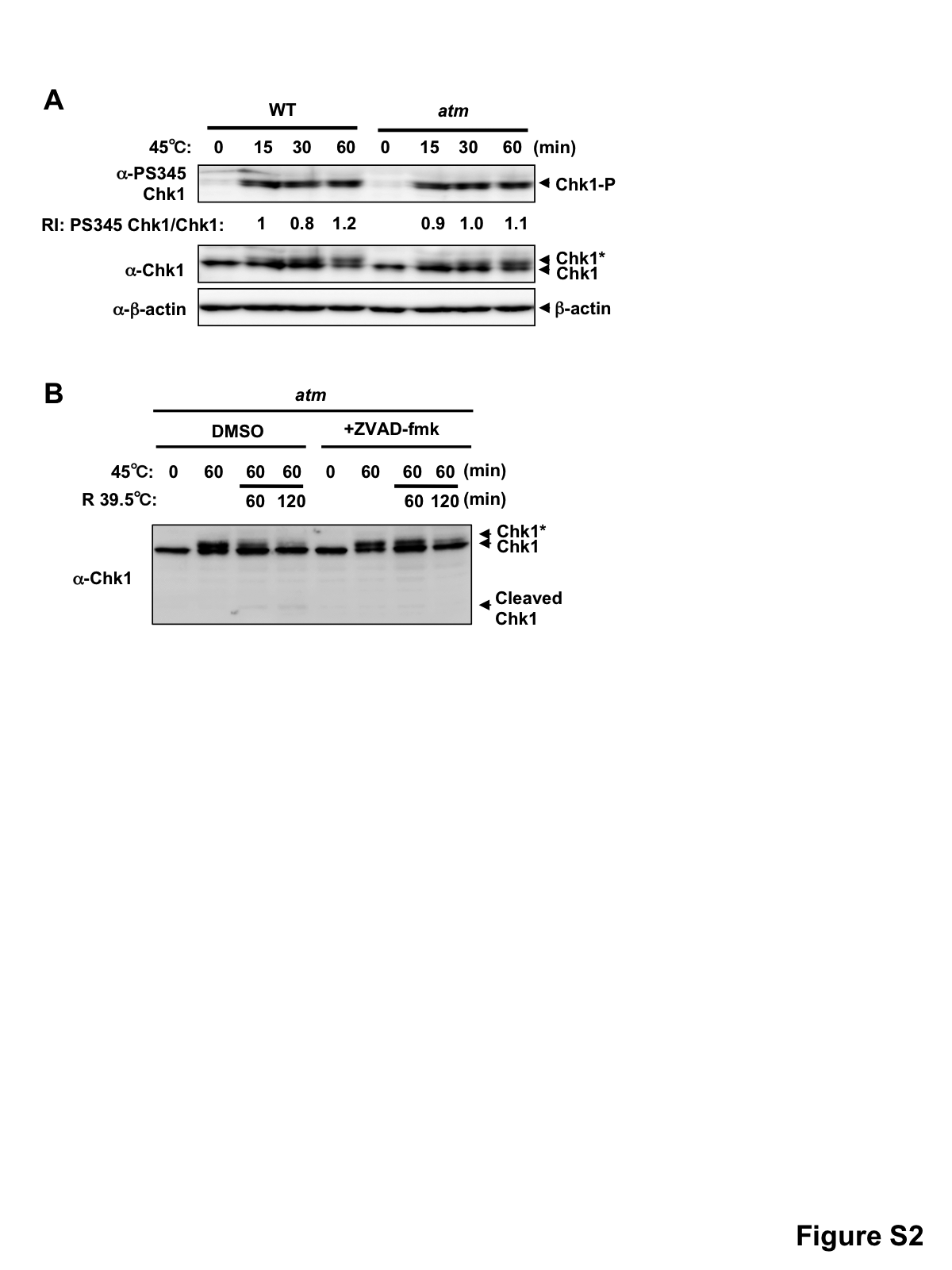

Supplement: Figure S2 — Cellular response to heat in ATM- deficient DT40 cells. A. Western blot. Wild-type (WT) and ATM-deficient (atm) DT40 cells were cultured at 45°C for the indicated time. RI: relative intensity compared to the sample of 45°C for 15 minutes in WT DT40 cells. B. Disappearance of the cleaved Chk1 peptide following treatment with the caspase inhibitor, ZVAD-fmk. atm cells were cultured at 45°C for 60 minutes and at 39.5°C for the indicated time in the presence or absence of 50 µM ZVAD-fmk. (TIFF) [file pone.0055361.s002.tiff]

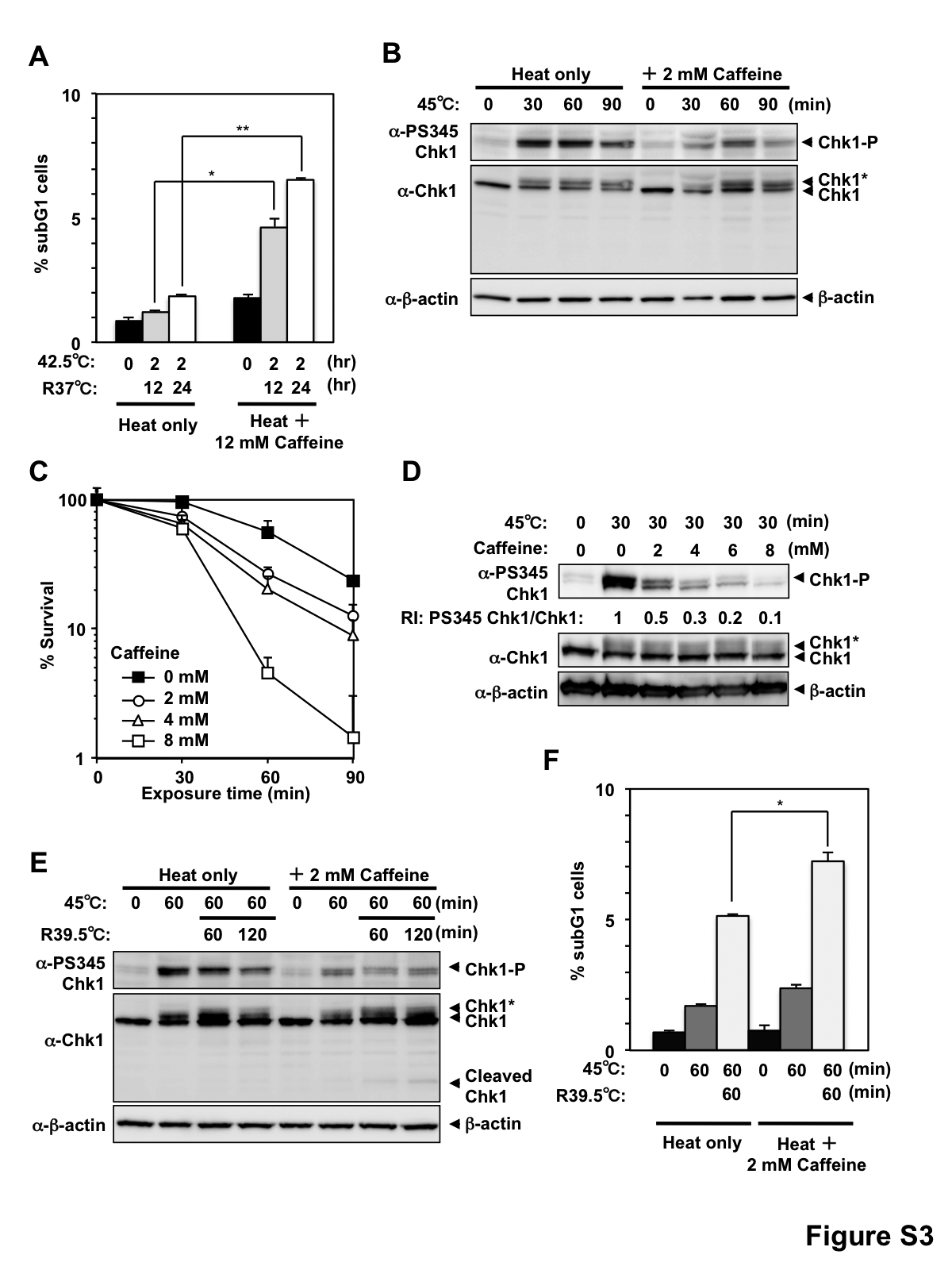

Supplement: Figure S3 — Caffeine enhanced heat cytotoxicity. A. SubG1 population. HeLa cells were cultured at 42.5°C for 2 hours and at 37°C for 12 or 24 hours in the presence or absence of 12 mM caffeine. *p = 0.0016, **p = 0.0002 (Student's t test). B. Western blot. Wild-type DT40 cells (WT) were cultured at 45°C for the indicated time in the presence or absence of 2 mM caffeine. C. Clonogenic survival. WT DT40 cells were cultured at 45°C for the indicated time in the presence of various concentration of caffeine. D. Western blot. WT DT40 cells were cultured at 45°C for 30 minutes in the presence of various concentration of caffeine. RI: relative intensity compared to the sample of 45°C for 30 minutes without caffeine in WT DT40 cells. E. Western blot. WT DT40 cells were cultured at 45°C for 60 minutes and at 39.5°C for the indicated time in the presence or absence of 2 mM caffeine. F. SubG1 population. WT DT40 cells were cultured at 45°C for 60 minutes and at 39.5°C for 60 minutes in the presence or absence of 2 mM caffeine. *p = 0.0069 (Student's t test). (TIFF) [file pone.0055361.s003.tiff]

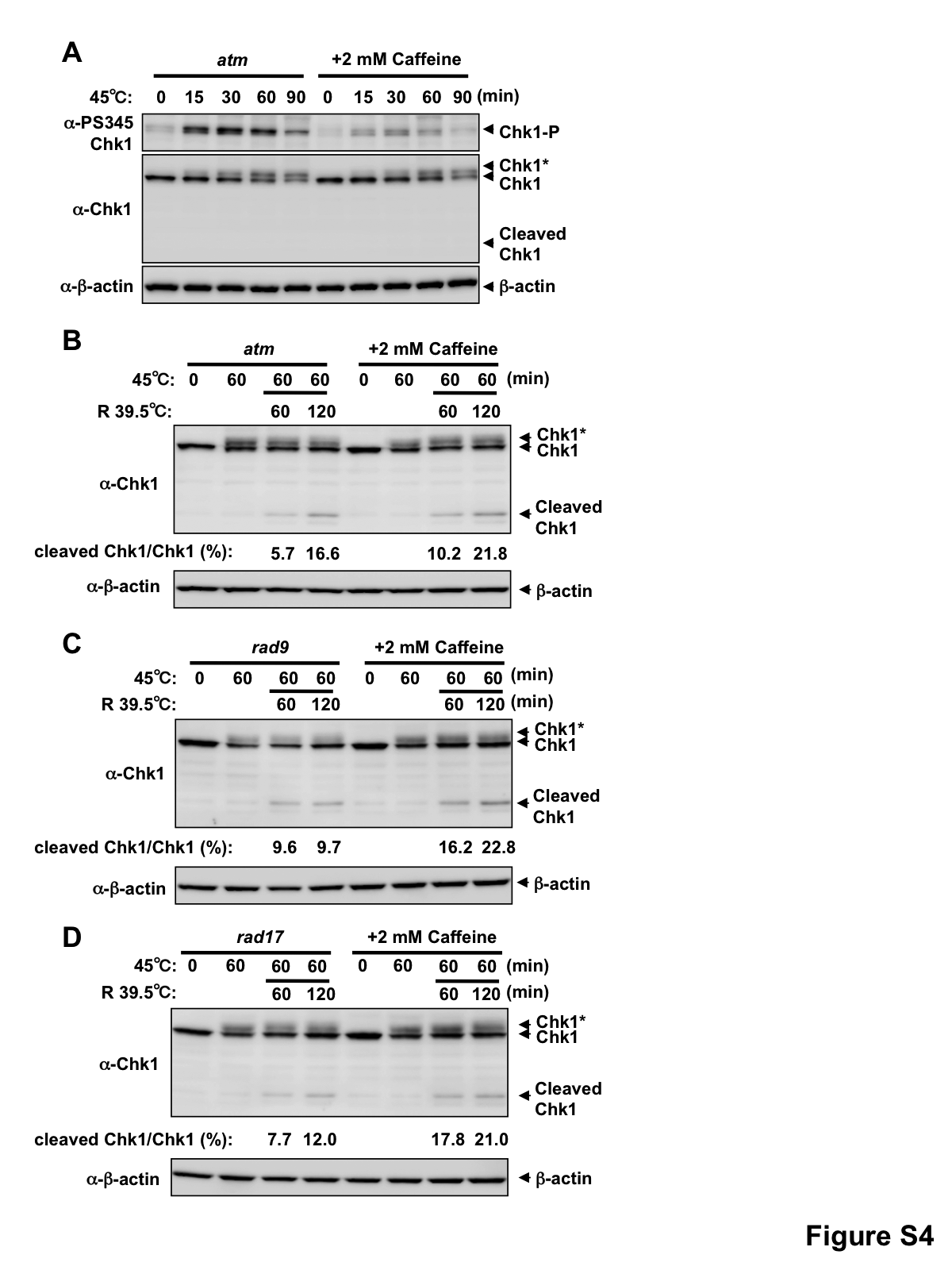

Supplement: Figure S4 — Cellular response to heat in the presence of caffeine in mutant DT40 cells. A. Western blot. ATM-deficient DT40 cells (atm) were cultured at 45°C for the indicated time in the presence or absence of 2 mM caffeine. B–D. Western blot. atm (B), rad9 (C) and rad17 (D) DT40 cells were cultured at 45°C for 60 minutes and at 39.5°C for the indicated time in the presence or absence of 2 mM caffeine. The percentage of cleaved Chk1 peptide per total. (TIFF) [file pone.0055361.s004.tiff]
